# PLIN5 Protects Against Ang II‐Induced Podocyte Lipotoxicity by Interacting With FKBP8 and Preserving Lipid Droplet–Mitochondria Contact

**DOI:** 10.1111/cpr.70257

**Published:** 2026-06-30

**Authors:** Ping Wang, Wenjie Chen, Jingjing Ke, Hongtu Hu, Zhuan Peng, Yue Qi, Guohua Ding, Jijia Hu

**Affiliations:** ^1^ Division of Nephrology Renmin Hospital of Wuhan University Wuhan Hubei China; ^2^ Nephrology and Urology Research Institute of Wuhan University Wuhan Hubei China; ^3^ Division of Nephrology Jingmen Central Hospital Affiliated to Jingchu University of Technology Jingmen Hubei China

**Keywords:** angiotensin II, FK506 binding protein 8, lipid droplet, lipotoxicity, perilipin 5, podocyte

## Abstract

Chronic kidney disease (CKD) remains a major global health challenge. Angiotensin II (Ang II)‐induced lipotoxicity is an important contributor to podocyte injury. Perilipin 5 (PLIN5) is a lipid droplet‐associated protein that helps maintain cellular metabolic homeostasis. However, how PLIN5 protects podocytes from lipotoxic stress remains incompletely understood. In this study, we generated podocyte‐specific PLIN5 knockout mice using the Cre‐loxP system and induced PLIN5 overexpression in vivo and in vitro. We found that Ang II markedly downregulated PLIN5 expression in podocytes both in vivo and in vitro. Podocyte‐specific deletion of PLIN5 aggravated Ang II‐induced lipid accumulation, mitochondrial dysfunction and apoptosis, whereas PLIN5 overexpression alleviated these abnormalities. Proteomic screening identified FK506‐binding protein 8 (FKBP8), an outer mitochondrial membrane protein, as a PLIN5‐interacting partner. Co‐immunoprecipitation and proximity ligation assays showed that the PLIN5‐FKBP8 interaction was reduced under Ang II stimulation. Functionally, FKBP8 knockdown disrupted lipid droplet‐mitochondria contact and exacerbated Ang II‐induced podocyte lipotoxicity. Domain‐mapping and rescue experiments further demonstrated that the 70–200 amino acid region of FKBP8 is required for PLIN5 binding and for preservation of lipid droplet‐mitochondria contact under lipotoxic stress. In addition, disruption of the PLIN5‐FKBP8 axis was associated with impaired fatty acid utilisation and altered mitochondrial homeostasis. Collectively, these findings support a model in which PLIN5 protects podocytes, at least in part, by interacting with FKBP8 and preserving lipid droplet‐mitochondria contact, thereby limiting Ang II‐induced lipotoxic injury.

## Graphical abstract

PLIN5‐FKBP8 tethers lipid droplets to mitochondria to counteract Ang II‐induced lipotoxicity.

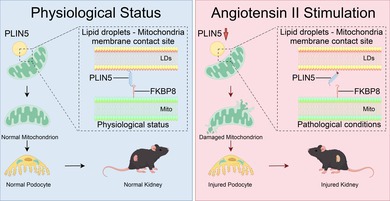

## 1 Introduction

Chronic kidney disease (CKD) poses a substantial global health burden, which is associated with severe adverse outcomes such as cardiovascular disease (CVD), kidney failure, premature mortality and diminished quality of life. The global prevalence of CKD is projected to become the fifth leading cause of mortality worldwide by 2040 [1, 2]. In recent years, treatment strategies targeting the renin‐angiotensin system (RAS) are cornerstone approaches in the management of CKD. A substantial body of clinical evidence has demonstrated that, beyond their blood pressure‐lowering effects, RAS inhibitors also effectively reduce proteinuria and slow the progression of kidney diseases [3]. However, the overall efficacy of RAS inhibitors in CKD remains suboptimal and a significant proportion of patients continue to progress to end‐stage kidney disease despite treatment [4, 5].

Angiotensin II (Ang II), as a central component of the RAS, plays a crucial role in several molecular pathophysiological processes, including inflammation and oxidative stress, which contribute to hypertension, extracellular matrix (ECM) remodelling and kidney fibrosis [6, 7]. The local production of Ang II can damage podocytes independently of the hemodynamic factors, representing a critical mechanism in the pathogenesis of Ang II‐induced CKD. Consequently, elucidating the underlying mechanisms has long been a major focus in nephrology [8].

Lipid droplets, also referred to as liposomes or oil bodies, are spherical organelles that function as the primary storage sites for neutral lipids (including cholesterol esters and triglycerides) in mammalian cells [9]. Free fatty acids (FFA) and their metabolites act as the main lipotoxic mediators. The perception and response of lipid droplets to FFA represent the core steps in avoiding lipotoxic damage [10]. The normal physical contact between lipid droplets and mitochondria is of great significance for efficient cellular lipid metabolism. Under specific pathological conditions, the disruption of this interaction causes the intracellular accumulation of free lipids, such as FFAs, which may potentially lead to lipotoxicity [11].

Perilipin5 (PLIN5), localised on the surface of lipid droplets, prevents lipase‐mediated degradation of droplets and serves as a key regulatory protein in lipid metabolism, playing a vital role in maintaining metabolic homeostasis [9]. A recent study has indicated that PLIN5 functions as a structural component of the membrane contact site complex, mediating the exchange of substances and information between lipid droplets and mitochondria [12]. Studies of the heart and skeletal muscles have shown that downregulation or functional impairment of PLIN5 expression leads to reduced lipid droplet‐mitochondria contact, decreased mitochondrial abundance and intracellular accumulation of free lipids. These changes may subsequently trigger inflammatory responses, endoplasmic reticulum stress and lipotoxic damage [13, 14].

Our previous studies showed that Ang II promotes free‐lipid accumulation in podocytes and disrupts lipid droplet metabolism, thereby driving lipotoxicity and accelerating kidney injury progression [15, 16, 17]. Recent evidence also indicates that PLIN5 contributes to podocyte lipotoxic injury, but the underlying mechanism remains incompletely understood [18]. These findings suggest that PLIN5 is involved in Ang II‐induced lipotoxicity in podocytes. However, direct evidence supporting this hypothesis is still limited. The present study aimed to investigate the role of PLIN5 in Ang II‐induced podocyte lipotoxicity and to determine whether PLIN5 acts by modulating lipid droplet–mitochondria contact. We further sought to identify the mitochondrial binding site for PLIN5.

## 2 Materials and Methods

### 2.1 Animal Studies

All animal experimental protocols were approved by the Animal Care Committee of Renmin Hospital of Wuhan University. The 8‐week‐old mice were randomly assigned to experimental groups and housed in a temperature‐controlled facility with a 12‐h light–dark cycle, with ad libitum access to food and water. All cages, bedding and water bottles were sterilised by autoclaving and cage changes were conducted three times per week. All procedures involving animals were performed in a blinded manner to minimise experimental bias. Blood samples and 24‐h urine collections were collected from the mice for biochemical analysis. After euthanasia, kidney tissues were harvested for histopathological evaluation. Total protein and RNA were subsequently extracted from the kidney tissues for further molecular investigations.

### 2.2 Mouse Model of Ang II Infusion

Eight‐week‐old male C57BL/6J mice were maintained under standard environmental conditions, including controlled humidity and temperature. The animals were randomly assigned to receive either normal saline or Ang II (700 ng/kg/min; Sigma, USA) via subcutaneously implanted osmotic minipumps for a period of 28 days.

### 2.3 Construction of Podocyte‐Specific PLIN5 Knockout Mice

PLIN5‐floxed mice (PLIN5^flox/+^, Hanheng Biotechnology Co. Ltd., Shanghai, China) on a C57BL/6J genetic background were utilised. To generate podocyte‐specific PLIN5 knockout mice, PLIN5‐floxed mice were bred with NPHS2‐Cre transgenic mice (B6.Cg‐Tg[NPHS2‐Cre], Bar Harbour, USA). Homozygous floxed mice lacking the Cre transgene served as the control group. The following primer sequences were used for genotyping:

– Forward primer for Cre: CATATTGGCAGAACGAAAACGC
– Reverse primer for Cre: CCTGTTTCACTATCCAGGTTACGG
– Forward primer for flox: GGTCCTCTCTAGGTCCCTAA TTCT
– Reverse primer for flox: CAGTCAAGGGTTTCTTGTCA AGTC

Only male mice (8 weeks old; PLIN5^flox/flox^/NPHS2‐Cre + and PLIN5^flox/flox^/NPHS2‐Cre‐) were included in the experiment to minimise variability due to hormonal cycles. Mice were administered either normal saline or Ang II (700 ng/kg/min; Sigma, USA) via subcutaneously implanted osmotic pumps. The experimental groups were as follows: saline‐treated control group, Ang II‐treated control group, saline‐treated conditional knockout group (cKO) and Ang II‐treated cKO, with six to eight mice per group. Urine and blood samples were collected at baseline (before pump implantation) and every 2 weeks post‐implantation. After 4 weeks, mice were euthanised following perfusion with normal saline and kidneys were harvested for subsequent analyses.

### 2.4 Delivery of Adeno‐Associated Virus Vector Serotype 9 (AAV9) in the Kidney

To induce PLIN5 overexpression in PLIN5^flox/flox^/NPHS2‐Cre + mice, AAV9 vectors were obtained from Hanbio Tech (Shanghai, China). Following the establishment of the Ang II infusion mouse model, 8‐week‐old male PLIN5^flox/flox^/NPHS2‐Cre + mice and their PLIN5^+/+^NPHS2‐Cre + littermates were anaesthetised. Genomic particles at a concentration of 1 × 10^12^ vector genomes (vg)/mL of either AAV9‐PLIN5 (experimental group) or AAV9‐CMV‐null (control group, AAV9‐Ctrl) were injected into six distinct sites within the kidney cortex using a glass micropipette, with 10 μL administered at each site.

### 2.5 Cell Culture and Transfection

Conditionally immortalised human podocytes were kindly provided by Professor Moin A. Saleem from Southmead Hospital, Bristol, UK. Cells were cultured at 33°C in RPMI 1640 medium (HyClone, USA) supplemented with 10% heat‐inactivated foetal bovine serum (Gibco, USA), 100 U/mL penicillin G (Invitrogen, USA) and insulin‐transferrin‐selenium (ITS, Invitrogen, USA). To induce differentiation, podocytes were cultured in ITS‐free medium at 37°C for 10–14 days. Differentiated human podocytes (HPC) were then treated with Ang II (10^−6^ M, Sigma, USA) or normal saline.

For siRNA transfection, small interfering RNAs targeting PLIN5 or FKBP8 and scrambled control RNA (Sangon Biotech, China) were used. Podocytes were transfected with 10 nM siRNA using HiPerFect transfection reagent (Qiagen, Germany) for 24 h, according to the manufacturer's instructions.

For plasmid transfection, pcDNA3.1‐PLIN5 (Paiwei Biotechnology, China), Flag‐tagged PLIN5 and HA‐tagged FKBP8 constructs, including full‐length FKBP8 (FKBP8‐FL), FKBP8‐Δ70–200 and FKBP8‐Δ280–320, were introduced into podocytes using X‐tremeGENE transfection reagent (Roche Diagnostics, Germany) according to the manufacturer's protocol. FKBP8 deletion mutants were cloned into the pcDNA3.1(+) vector (Genecefe Biotechnology Corporation, Wuxi, China). For rescue experiments, podocytes were first transfected with si‐FKBP8 or control siRNA for 24 h, followed by transfection with empty vector or the indicated FKBP8 plasmids for an additional 24 h before Ang II stimulation. After transfection and treatment, cells were collected for subsequent analyses.

### 2.6 Urine Collection

The mice were individually housed in metabolic cages and urine samples were collected over a 24‐h period. Following collection, the samples were centrifuged at 1000*g* for 10 min at 4°C. The resulting supernatant was then stored at −80°C for subsequent biochemical analysis.

### 2.7 Histology and Immunohistochemical (IHC) Staining

Kidney tissues were systemically perfused with ice‐cold phosphate‐buffered saline (PBS) followed by 4% paraformaldehyde (PFA; pH 7.4). Following tissue harvest, the kidneys were processed according to standard histological protocols. In brief, the tissues were embedded in paraffin, sectioned into 4‐μm‐thick slices and stained with haematoxylin and eosin (H&E) as well as periodic acid‐Schiff (PAS). For each mouse, at least 10 glomeruli were captured at a magnification of 40× using an Olympus microscope (Tokyo, Japan). The positively stained areas within the glomeruli were quantified using ImageJ software (National Institutes of Health, USA). For IHC staining, the sections were incubated with the corresponding primary antibody. Subsequently, histochemical intensity was assessed in 10 randomly selected fields from each sample using ImageJ and the average intensity was calculated as a representative data point for each sample.

### 2.8 Immunofluorescence Analysis and Proximity Ligation Assay (PLA)

Immunofluorescence analysis was conducted on frozen kidney sections from human and mouse tissues, as well as on cultured cells. Following dewaxing and antigen retrieval in paraffin sections, the samples were blocked with 5% bovine serum albumin (BSA) for 30 min. Subsequently, the samples were incubated overnight at 4°C with the corresponding primary antibodies. On the following day, the sections or cell slides were incubated with fluorescent secondary antibodies (Antgene, China) for 1 h in the dark. Nuclear staining was carried out using 4′,6‐diamidino‐2‐phenylindole (DAPI) (Antgene, China). Fluorescence images were captured and analysed using a confocal microscope (Olympus, Japan).

For analysis of PLIN5 expression in kidney tissues and cultured podocytes, primary antibodies against PLIN5 and Wilms tumour protein 1 (WT‐1) or synaptopodin were used as indicated. For colocalisation analysis, podocytes grown on glass coverslips were incubated with primary antibodies against PLIN5, FKBP8 or TOMM20, followed by the corresponding fluorescent secondary antibodies. Neutral lipid droplets were stained with BODIPY 493/503 as described below. Images were acquired using identical laser intensity, gain and exposure settings within the same experiment. Colocalisation was quantified using ImageJ software (National Institutes of Health, USA) with the JACoP plugin. Pearson's correlation coefficient or Manders' overlap coefficient was calculated from at least five randomly selected fields per group.

To further evaluate the interaction between PLIN5 and FKBP8 in situ, a PLA was performed using a Duolink In Situ PLA kit (Sigma, USA) according to the manufacturer's instructions. Briefly, differentiated podocytes grown on glass coverslips were treated with saline or Ang II, fixed with 4% paraformaldehyde for 15 min and permeabilised with 0.1% Triton X‐100 for 10 min. After blocking, the cells were incubated overnight at 4°C with primary antibodies against PLIN5 and FKBP8 derived from different host species. No‐primary‐antibody controls were processed in parallel. PLA probes, ligation and amplification were then performed according to the manufacturer's protocol and nuclei were counterstained with DAPI. PLA signals were visualised by confocal microscopy (Olympus, Japan). PLA puncta per cell were quantified using ImageJ software in at least 30–50 cells per group from each of three independent experiments. Cytoplasmic and perinuclear localisation was assessed using DAPI counterstaining and enlarged confocal views. All image analyses were performed in a blinded manner.

### 2.9 Transmission Electron Microscopy (TEM)

The kidney cortex was post‐fixed with 1% osmium tetroxide, dehydrated in a graded ethanol series and embedded in Epon epoxy resin. Ultrathin sections (70–90 nm) were cut using an ultramicrotome, stained with uranyl acetate and lead citrate. The prepared samples were then examined under a transmission electron microscope (Hitachi, Japan). For each mouse, five glomeruli were analysed and 10 micrographs were captured per glomerulus.

### 2.10 Western Blotting Analysis

Protein samples were extracted from glomerular or podocyte lysates under various experimental conditions using RIPA lysis buffer (Beyotime, China), supplemented with a protease inhibitor cocktail (Sigma, USA), phenylmethylsulfonyl fluoride (PMSF, Beyotime, China) and phosphatase inhibitors (Beyotime, China). The extracted proteins were separated by electrophoresis on a 10% SDS‐PAGE gel and subsequently transferred onto a PVDF membrane (Millipore, USA). The membrane was blocked with 5% skim milk at room temperature for 1 h, followed by incubation with primary antibodies overnight at 4°C. After three washes with TBST buffer, the membrane was incubated with an HRP‐conjugated secondary antibody at room temperature for 1 h. Protein bands were detected using an enhanced chemiluminescence (ECL) detection kit (Servicebio, China) and visualised using a Bio‐Rad chemiluminescence imaging system (Bio‐Rad, USA). The expression levels of target proteins were quantified using ImageJ software.

In addition to PLIN5 and FKBP8, proteins related to fatty acid oxidation and mitochondrial quality control, including CPT1α, p‐ACC/ACC, PINK1 and Parkin, were analysed under the indicated experimental conditions. For transfection‐based experiments, Western blotting was also used to verify the efficiency of PLIN5 or FKBP8 knockdown, as well as overexpression of PLIN5 or FKBP8 constructs. Protein expression levels were normalised to β‐actin. The antibodies used in this study are described in Table S1.

### 2.11 Co‐Immunoprecipitation (Co‐IP)

Co‐IP was employed to confirm the interaction between PLIN5 and FKBP8. Podocytes were lysed in immunoprecipitation (IP) buffer supplemented with protease inhibitors (Sigma, USA). A volume of 400 μL of the resulting cell lysate was incubated with 40 μL of protein A/G agarose beads at 4°C for 2 h. Following centrifugation, the supernatant was incubated with either anti‐PLIN5 antibody, anti‐FKBP8 antibody anti‐HA antibody or control IgG at 4°C overnight. The beads were subsequently washed three times with IP buffer and the bound proteins were eluted by boiling in loading buffer. The eluted protein samples were analysed for the presence of PLIN5 and FKBP8 using Western blotting.

To determine whether the interaction between PLIN5 and FKBP8 was altered under disease‐relevant conditions Co‐IP was additionally performed in podocytes treated with saline or Ang II. Equal amounts of protein lysates were processed in parallel and input lysates were analysed simultaneously. For experiments involving deletion mutants, podocytes were transfected with Flag‐tagged PLIN5 together with HA‐tagged full‐length FKBP8 (FKBP8‐FL), FKBP8‐Δ70–200 or FKBP8‐Δ280–320 constructs. Cell lysates were immunoprecipitated with anti‐HA antibody and analysed by immunoblotting for HA and Flag‐PLIN5. Band intensities of Co‐IP proteins were quantified using ImageJ software and normalised to the corresponding immunoprecipitated protein levels where indicated.

### 2.12 Glomerulus Isolation

The glomeruli were isolated using a modified sieving method. Prior to kidney removal, the organ was perfused with sterile Hank's Balanced Salt Solution (HBSS). The kidney tissue was then excised and manually minced into approximately 1 mm^3^ fragments using a scalpel. These fragments were subsequently digested in an enzymatic solution containing collagenase II (#10103586001, Roche), protease E (#P6911, Sigma) and deoxyribonuclease I (#D4527, Sigma) at 37°C for 30 min. Following digestion, the sample was sequentially filtered through 100‐μm (BD, USA), 70‐μm (BD, USA) and 40‐μm (Greiner bio‐one, Germany) cell strainers. The glomeruli were retained on the inner surface of the 40‐μm cell strainer. Finally, the collected glomeruli were transferred into a centrifuge tube, centrifuged at 200*g* for 5 min at 4°C and the resulting pellet was resuspended in 5 mL of culture medium.

### 2.13 Isolation of Primary Podocytes

Primary podocytes were isolated from double‐fluorescent Cre reporter mice. Glomeruli were isolated from two‐week‐old mT/mG/NPHS2‐Cre mice, generated by breeding B6.Cg‐Tg(NPHS2‐Cre)295Lbh/J mice (Jackson Laboratory; catalogue number #008205) with Gt(ROSA)26Sortm4(ACTB‐tdTomato,‐EGFP)Luo/J mice (Jackson Laboratory; catalogue number #007676). Subsequently, the glomeruli were cultured in standard podocyte medium, consisting of RPMI 1640 supplemented with 10% foetal bovine serum, 100 μg/mL streptomycin and 1× ITS. Following 5 days of expansion, green fluorescent protein (GFP)‐positive cells (primary podocytes) were separated from GFP‐negative cells (non‐podocyte cells) using flow cytometry (BD, USA). The isolated cells were then maintained at 37°C in a 5% CO₂ incubator.

### 2.14 Detection of Apoptosis

The apoptosis of podocytes was assessed using flow cytometry. Briefly, apoptotic cells were quantified using the BD Pharmingen PE Annexin V Apoptosis Detection Kit I (BD Biosciences, USA), following the manufacturer's instructions.

### 2.15 Analysis of Reactive Oxygen Species (ROS) and Adenosine Triphosphate (ATP)

Podocytes were cultured in six‐well plates. According to the manufacturer's instructions:

1. The ROS levels in podocytes were evaluated by flow cytometry using a ROS detection kit (#S0033S, Beyotime, China). Cells were harvested by trypsinisation and resuspended in binding buffer. After a 30‐min incubation at 37°C in the dark, the cells were stained. Fluorescence was measured using a NovoCyte flow cytometer (Acea Biosciences) and data were analysed with FlowJo software.
2. ATP levels were determined using the ATP Detection Kit (#S0027, Beyotime, China) and quantification was performed using a microplate reader.

### 2.16 Oxygen Consumption Rate (OCR) Measurements

The OCR measurements were performed to assess mitochondrial function by quantifying the OCR and extracellular acidification rate (ECAR) using the XFe96 extracellular flux analyser (Seahorse Bioscience, USA) according to the manufacturer's instructions. Briefly, cells were seeded in XF 24‐well cell culture microplates and OCR was measured following sequential administration of oligomycin, FCCP, rotenone and antimycin A.

### 2.17 MitoTracker Red Staining

The morphology of mitochondria was assessed using MitoTracker Red CMXRos staining (#M7512, Invitrogen, USA) following the manufacturer's instructions. Fixed cell slides were incubated with 50 nM MitoTracker Red dye at 37°C for 30 min. Fluorescence images were captured using an Olympus confocal microscope.

### 2.18 BODIPY 493/503 Fluorescent Staining

To stain neutral lipids in the cytoplasm, BODIPY 493/503, a commonly used fluorescent dye, was employed. Cell slides were fixed with 4% paraformaldehyde and then incubated with BODIPY 493/503 (4,4‐difluoro‐1,3,5,7,8‐pentamethyl‐4‐bora‐3a,4a‐diaza‐s‐indacene, 1 μM, D3922, ThermoFisher, USA) in the dark for 1 h. Following removal of the excess dye, the cells were counterstained with DAPI Fluoromount (ANT063, China) for 15 min, air‐dried and mounted. The slides were then examined using an Olympus confocal fluorescence microscope (Olympus, Japan). For frozen kidney sections, following fixation and blocking, the sections were incubated with WT1 antibody (BM4216, Boster, China) at 37°C for 1 h, followed by incubation with secondary antibody and BODIPY dye. Finally, the sections were counterstained with DAPI.

### 2.19 Structure‐Based Protein Interaction

The protein structures of PLIN5 and FKBP8 were analysed using SWISS‐MODEL (https://www.swissmodel.expasy.org), a template‐based homology modelling tool. The potential interaction interfaces between PLIN5 and FKBP8 were predicted using the PRISM tool (http://cosbi.ku.edu.tr/prism), based on their structural data obtained from the Protein Data Bank. The predicted structural model was used to identify potential PLIN5‐binding regions within FKBP8.

To experimentally verify the predicted binding regions, Flag‐tagged PLIN5 and HA‐tagged FKBP8 constructs, including full‐length FKBP8 (FKBP8‐FL), FKBP8‐Δ70–200 and FKBP8‐Δ280–320, were cloned into the pcDNA3.1(+) vector (Genecefe Biotechnology Corporation, Wuxi, China). For rescue experiments, siRNA‐resistant FKBP8 constructs were generated by introducing silent mutations into the FKBP8 siRNA‐targeting sequence using a site‐directed mutagenesis kit (Sigma, USA), without altering the encoded amino acid sequence. All constructs were verified by Sanger sequencing before use.

For domain‐mapping and rescue assays, podocytes were transfected with the indicated plasmids using X‐tremeGENE transfection reagent (Roche Diagnostics, Germany) according to the manufacturer's instructions. In rescue experiments, cells were first transfected with si‐FKBP8 or control siRNA for 24 h, followed by transfection with empty vector, siRNA‐resistant FKBP8‐FL, FKBP8‐Δ70–200 or FKBP8‐Δ280–320 for an additional 24 h before Ang II stimulation. After treatment, the cells were subjected to Co‐IP, immunofluorescence, BODIPY staining, flow cytometry, Seahorse analysis, ATP measurement and biochemical assays as indicated.

### 2.20 Statistical Analysis

All experiments were conducted at least three times. Data are expressed as mean ± standard deviation (SD). Statistical analysis was performed under the assumption of normal data distribution using GraphPad Prism 9.0 (GraphPad Software, USA). For comparisons between two independent groups, an unpaired, two‐tailed Student's *t*‐test was applied. One‐way analysis of variance (one‐way ANOVA), followed by Tukey's post hoc test, was used to assess differences among multiple groups with a single variable. For comparisons involving multiple variables across groups, two‐way analysis of variance (two‐way ANOVA) combined with Tukey's post hoc test was employed. When data did not follow a Gaussian distribution, the Kruskal–Wallis test was used, followed by Dunn's post hoc test for multiple comparisons. The Spearman correlation coefficient was calculated to evaluate the relationship between two continuous variables. A *p*‐value < 0.05 was considered statistically significant. Detailed sample sizes are indicated in the figure legends.

## 3 Results

### 3.1 Ang II‐Induced Alterations in Kidney Pathology, Lipid Metabolism and PLIN5 Expression in Animal Model

**FIGURE 1:**
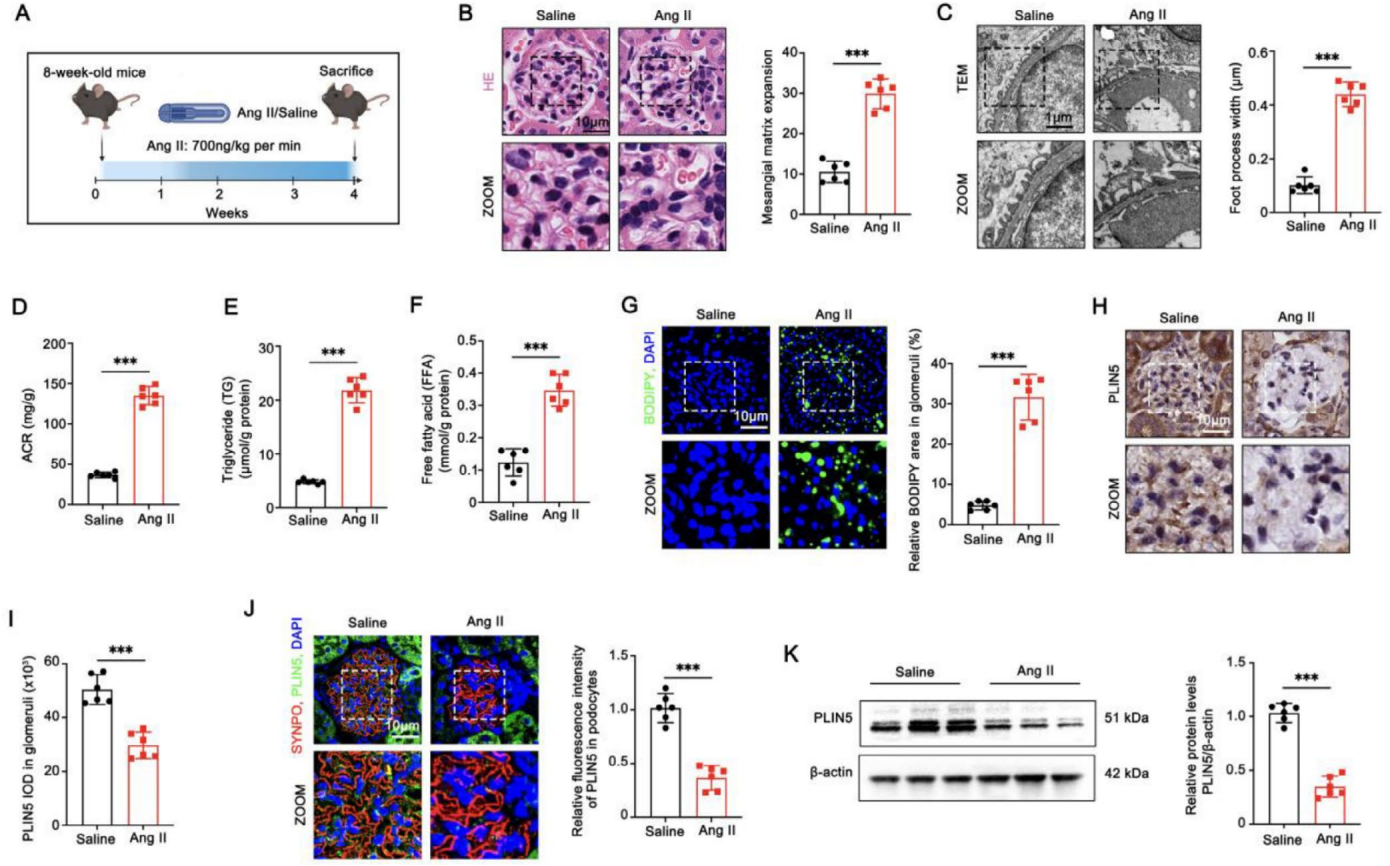
Ang II‐induced alterations in kidney pathology, lipid metabolism and PLIN5 expression in animal model. (A) Schematic of the experimental protocol. Eight‐week‐old mice were implanted with osmotic minipumps delivering Ang II (700 ng/kg per min) or saline for 4 weeks and then sacrificed. (B) Representative HE‐stained glomerular sections from saline‐ and Ang II‐treated mice and quantification of mesangial matrix expansion. Scale bar, 10 μm. (C) Representative transmission electron microscopy (TEM) images showing podocyte foot processes in saline‐ and Ang II‐treated mice and quantification of foot process width. Scale bar, 1 μm. (D–F) Urinary albumin‐to‐creatinine ratio (ACR; D), renal triglyceride (TG; E) and free fatty acid (FFA; F) contents in saline‐ and Ang II‐treated mice. (G) Representative images of neutral lipid accumulation in glomeruli detected by BODIPY (green) with DAPI (blue) and quantification of the relative BODIPY‐positive area. Scale bar, 10 μm. (H) Representative immunohistochemical staining of PLIN5 in glomeruli from saline‐ and Ang II‐treated mice. Scale bar, 10 μm. (I) Quantification of glomerular PLIN5 expression presented as integrated optical density (IOD). (J) Representative confocal images of glomeruli co‐stained for synaptopodin (SYNPO, red) and PLIN5 (green) with DAPI (blue) and quantification of the relative fluorescence intensity of PLIN5 in SYNPO‐positive podocytes. Scale bar, 10 μm. (K) Representative Western blot and densitometric analysis of PLIN5 protein levels in kidney tissue with β‐actin as a loading control. Data are presented as mean ± SEM (*n* = 6 mice per group). ***p < 0.001 versus saline (unpaired two‐tailed Student's t‐test).

Ang II infusion was utilised to establish an Ang II‐induced kidney injury model in wild‐type mice. The model showed mesangial cell proliferation, extensive effacement of podocyte foot processes in the glomeruli (Figure 1A–C) and an elevated urine albumin‐to‐creatinine ratio (ACR) (Figure 1D). Furthermore, Ang II‐treated mice displayed marked lipid accumulation in the glomeruli, particularly within podocytes, along with significantly increased triglyceride (TG) and FFA levels compared to the saline‐treated group (Figure 1E,F). BODIPY staining further confirmed increased neutral lipid deposition in the glomeruli of Ang II‐treated mice (Figure 1G). IHC analyses of glomeruli revealed a significant reduction in PLIN5 expression (Figure 1H,I). Fluorescent co‐staining for PLIN5 and synaptopodin, a specific podocyte marker, showed markedly decreased PLIN5 signal in both glomerular regions and synaptopodin‐positive podocytes (Figure 1J). Western blotting combined with densitometric quantification further validated the downregulation of PLIN5 protein levels (Figure 1K). Collectively, these results indicate that PLIN5 may play a critical role in Ang II‐induced lipid accumulation and subsequent podocyte injury.

### 3.2 Lipid Accumulation, Lipotoxicity, Lipid Droplet‐Mitochondria Contact and PLIN5 Expression in Ang II‐Treated Podocytes In Vitro

**FIGURE 2:**
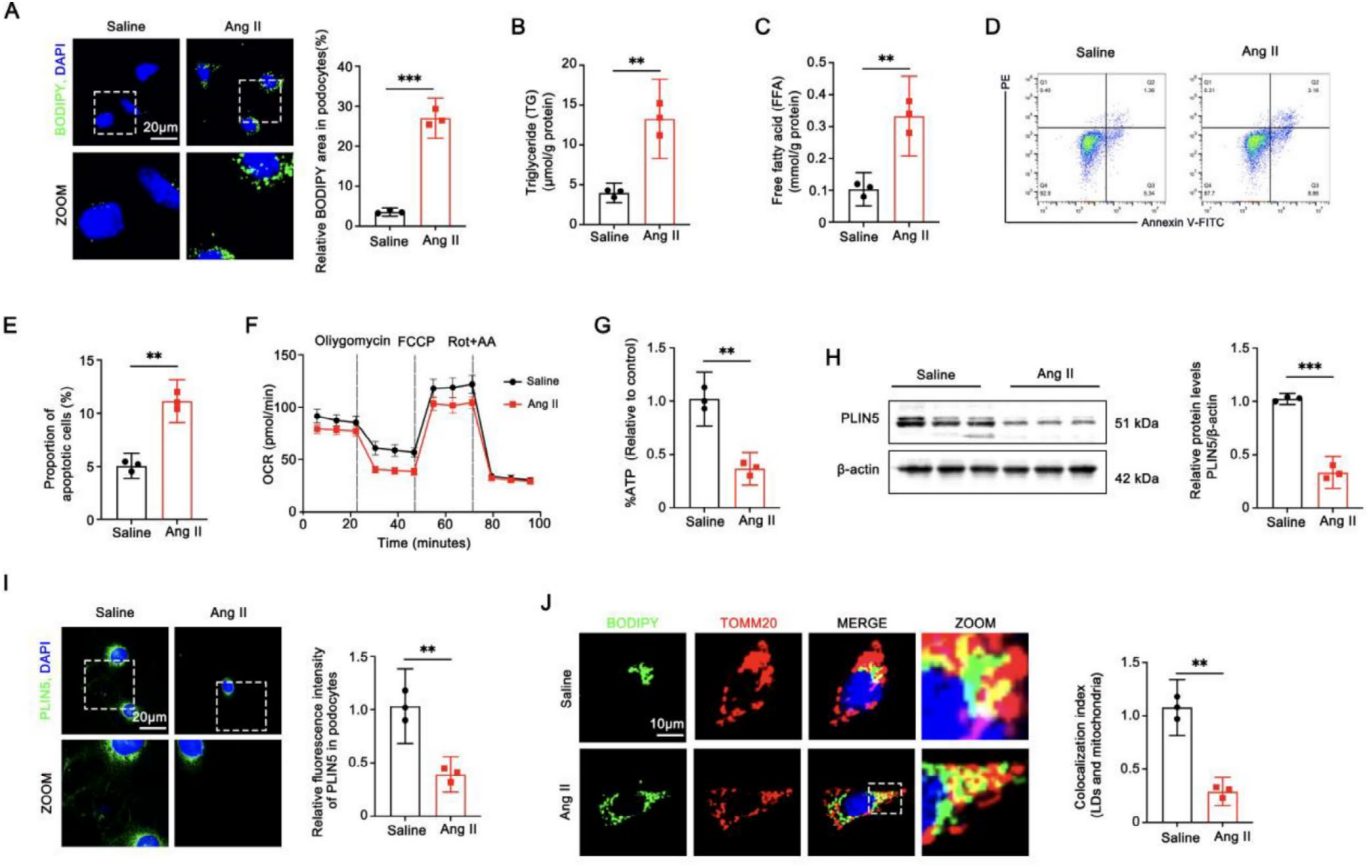
Lipid accumulation, lipotoxicity, lipid droplet–mitochondria contact and PLIN5 expression in Ang II‐treated podocytes in vitro. (A) Representative confocal images of BODIPY (green) and DAPI (blue) staining in cultured podocytes treated with saline or Ang II and quantification of the relative BODIPY‐positive area. Scale bar, 20 μm. (B, C) Cellular triglyceride (TG) and free fatty acid (FFA) contents in saline‐ and Ang II‐treated podocytes. (D, E) Representative flow‐cytometric dot plots of Annexin V‐FITC/PI staining (D) and quantification of the proportion of apoptotic podocytes (E). (F) Oxygen consumption rate (OCR) traces in podocytes measured by Seahorse analysis under basal conditions and after sequential addition of oligomycin, FCCP and rotenone/antimycin A (Rot + AA). (G) Intracellular ATP levels expressed as a percentage of control. (H) Representative Western blot and densitometric analysis of PLIN5 protein expression in podocytes, with β‐actin as a loading control. (I) Representative immunofluorescence images of PLIN5 (green) with DAPI (blue) and quantification of the relative fluorescence intensity of PLIN5 in podocytes. Scale bar, 20 μm. (J) Representative images showing lipid droplets (BODIPY, green) and mitochondria (TOMM20, red) in podocytes, merged and enlarged (ZOOM) views and quantification of the colocalisation index between lipid droplets and mitochondria. Scale bar, 10 μm. Data are presented as mean ± SEM from at least three independent experiments. **p < 0.01, ***p < 0.001 versus saline (unpaired two‐tailed Student's *t*‐test).

To validate our findings from the animal model, we cultured primary podocytes in vitro and stimulated them with Ang II. Consistent with the in vivo observations, Ang II treatment induced lipid accumulation in podocytes (Figure 2A–C), leading to lipotoxicity, as evidenced by increased apoptosis quantified via flow cytometry, reduced OCR profiles and decreased intracellular ATP levels (Figure 2D–G). These effects were accompanied by a significant downregulation of PLIN5 expression (Figure 2H,I). Furthermore, our analysis revealed a marked reduction in lipid droplet–mitochondria contact compared to control podocytes cultured under normal conditions (Figure 2J). These results indicate that Ang II‐induced PLIN5 downregulation is associated with increased lipid accumulation, lipotoxicity and impaired lipid droplet–mitochondria interaction in podocytes.

### 3.3 Podocyte‐Specific Deletion of PLIN5 Aggravated Ang II‐Induced Glomerular Lipid Dysregulation and Reduced Lipid Droplet‐Mitochondria Contact In Vivo

**FIGURE 3:**
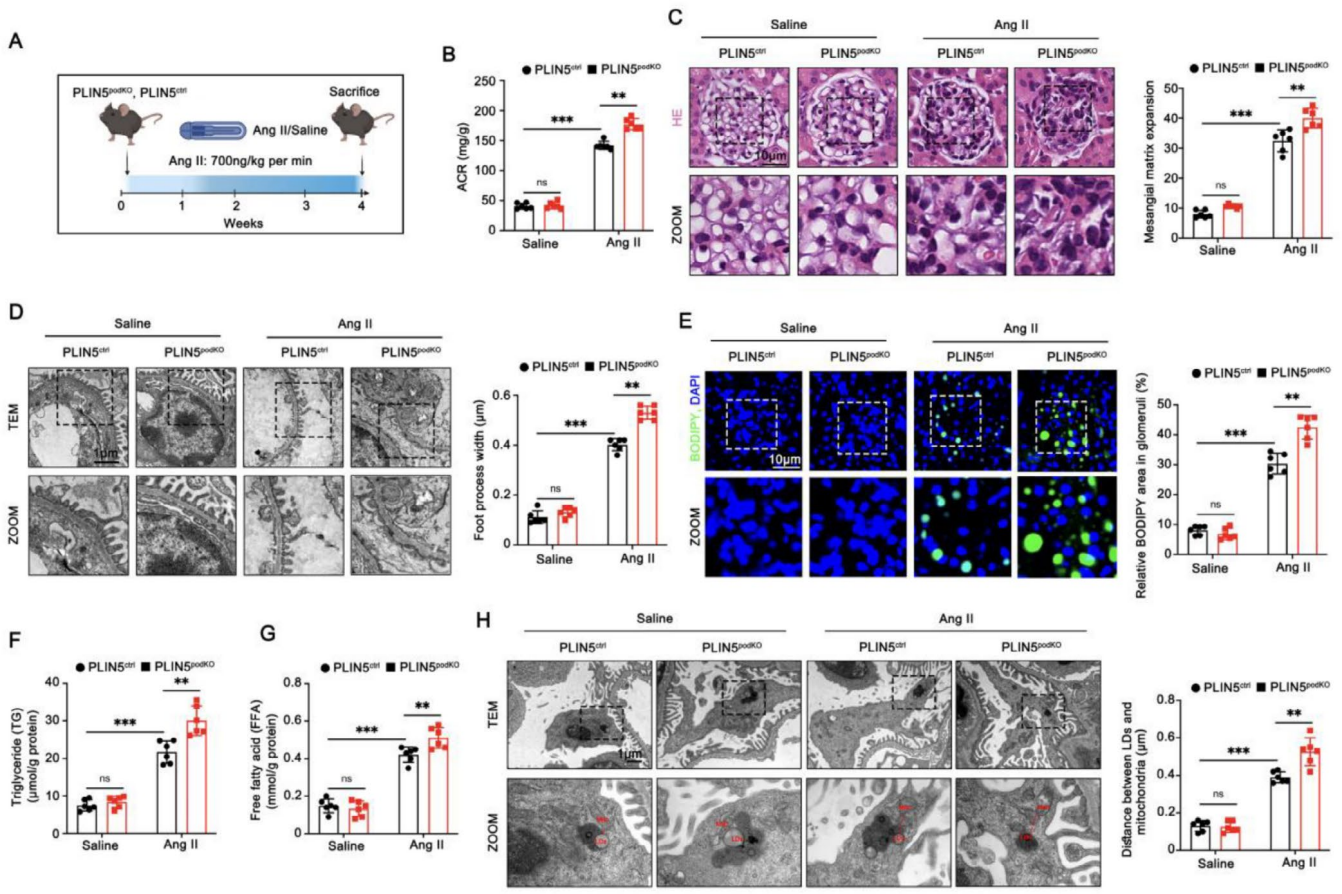
Podocyte‐specific deletion of PLIN5 aggravated Ang II‐induced glomerular lipid dysregulation and reduced lipid droplet‐mitochondria contact in vivo. (A) Experimental scheme. Podocyte‐specific PLIN5 knockout (PLIN5^podKO^) and littermate control (PLIN5^fl/fl^; PLIN5^Ctrl^) mice were implanted with osmotic minipumps delivering Ang II (700 ng/kg per min) or saline for 4 weeks and then sacrificed. (B) Urinary albumin‐to‐creatinine ratio (ACR) in the indicated groups. (C) Representative HE‐stained kidney sections showing glomerular morphology and quantification of mesangial matrix expansion. Scale bar, 10 μm. (D) Representative transmission electron microscopy (TEM) images of podocyte foot processes and quantification of foot process width. Scale bar, 1 μm. (E) Representative images of neutral lipid accumulation in glomeruli detected by BODIPY (green) with DAPI (blue) and quantification of relative BODIPY‐positive area. (F, G) Renal triglyceride (TG) (F) and free fatty acid (FFA) (G) contents in the indicated groups. (H) Representative TEM images showing lipid droplets (LDs) and mitochondria (Mt) in podocytes and quantification of the mean distance between LDs and mitochondria. Scale bar, 1 μm. Data are presented as mean ± SEM (*n* = 6 mice per group). ns, not significant; **p < 0.01, ***p < 0.001 (two‐way ANOVA with post hoc multiple comparisons).

To investigate the role of PLIN5 in Ang II‐induced lipotoxicity in podocytes, podocyte‐specific PLIN5 knockout (PLIN5^flox/flox^/NPHS2‐Cre+, PLIN5^podKO^) mice were generated using the Cre‐Loxp recombination system (Figure S1). The mice were implanted with osmotic minipumps delivering Ang II or saline for 4 weeks and then sacrificed (Figure 3A). Subsequently, we evaluated the effects of PLIN5 deletion on kidney morphology (Figure 3B–D), lipid accumulation (Figure 3E–G) and lipid droplet–mitochondria contact in podocytes (Figure 3H). As shown in the results, urinary ACR was significantly higher in PLIN5^flox/flox^/NPHS2‐Cre + mice than in PLIN5^flox/flox^/NPHS2‐Cre‐ mice. HE staining and TEM results revealed increased glomerulosclerosis and podocyte foot process effacement in the glomeruli of PLIN5‐specific knockout mice. Moreover, total TG and FFA levels were significantly increased, while the interaction between lipid droplets and mitochondria was reduced in PLIN5^flox/flox^/NPHS2‐Cre + mice compared to PLIN5^flox/flox^/NPHS2‐Cre‐ mice.

### 3.4 PLIN5 Knockdown Exacerbated Lipid Dysregulation and Lipid Droplet‐Mitochondria Contact Disorder in Podocytes Induced by Ang II

**FIGURE 4:**
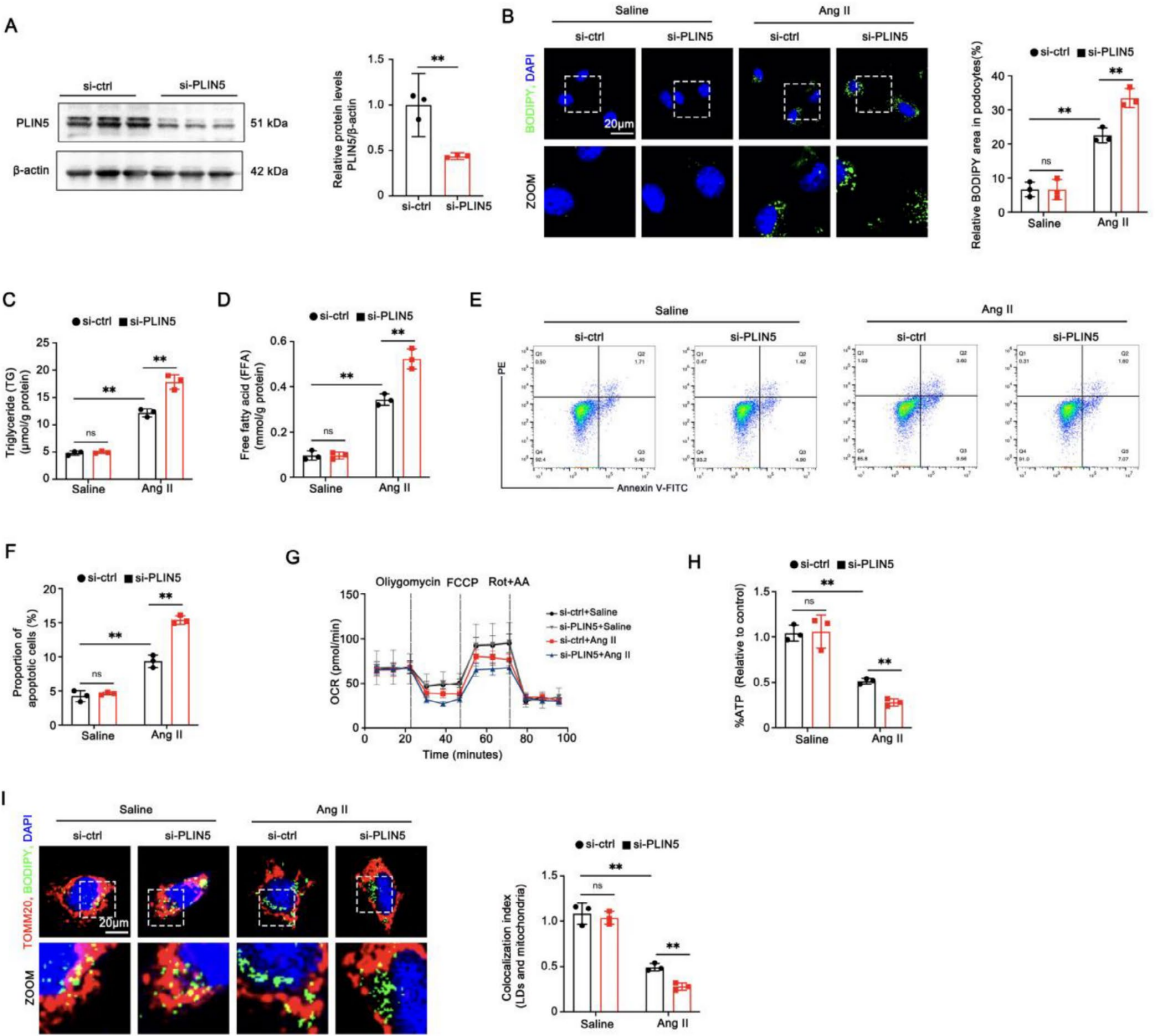
PLIN5 knockdown exacerbated lipid dysregulation and lipid droplet‐mitochondria contact disorder in podocytes induced by Ang II. (A) Validation of PLIN5 knockdown in cultured podocytes transfected with control siRNA (si‐ctrl) or PLIN5 siRNA (si‐PLIN5) by Western blot and densitometric analysis (β‐actin as loading control). (B) Representative confocal images of neutral lipid droplets stained with BODIPY (green) and nuclei stained with DAPI (blue) in si‐ctrl or si‐PLIN5 podocytes treated with saline or Ang II and quantification of the relative BODIPY‐positive area. Scale bar, 20 μm. (C, D) Cellular triglyceride (TG) (C) and free fatty acid (FFA) (D) contents in podocytes under the indicated conditions. (E, F) Representative Annexin V–FITC/PI flow‐cytometric plots (E) and quantification of the proportion of apoptotic podocytes (F). (G) Oxygen consumption rate (OCR) of podocytes measured by Seahorse analysis under basal conditions and after sequential injections of oligomycin, FCCP and rotenone/antimycin A (Rot + AA). (H) Intracellular ATP levels expressed as a percentage of saline‐treated si‐ctrl cells. (I) Representative confocal images showing lipid droplets (BODIPY, green), mitochondria (TOMM20, red) and nuclei (DAPI, blue) in si‐ctrl or si‐PLIN5 podocytes with or without Ang II stimulation, with enlarged ZOOM views and quantification of the colocalisation index between lipid droplets and mitochondria. Scale bar, 20 μm. Data are presented as mean ± SEM from at least three independent experiments. ns, not significant; **p < 0.01, ***p < 0.001 (two‐way ANOVA with post hoc multiple comparisons).

To further investigate the role of PLIN5 in podocyte lipotoxicity and its regulatory effect on lipid droplet‐mitochondria interactions, we transfected cultured podocytes with PLIN5‐specific small interfering RNA prior to Ang II treatment. As expected, PLIN5 protein expression was markedly reduced following transfection (Figure 4A). We then assessed Ang II‐induced lipid accumulation (Figure 4B–D), lipotoxicity (Figure 4E–H) and alterations in lipid droplet‐mitochondria contact (Figure 4I). The results demonstrated that Ang II treatment impaired mitochondrial metabolism and this dysfunction was exacerbated by PLIN5 knockdown. Moreover, PLIN5 deficiency significantly increased the proportion of Ang II‐induced apoptotic cells. These findings suggest that PLIN5 knockdown disrupts lipid droplet‐mitochondria coupling, resulting in enhanced lipid accumulation and lipotoxicity, thereby aggravating Ang II‐induced mitochondrial impairment and podocyte injury.

### 3.5 Overexpression of PLIN5 Alleviated Ang II‐Induced Lipid Dysregulation and Lipid Droplet‐Mitochondria Contact Disorder

**FIGURE 5:**
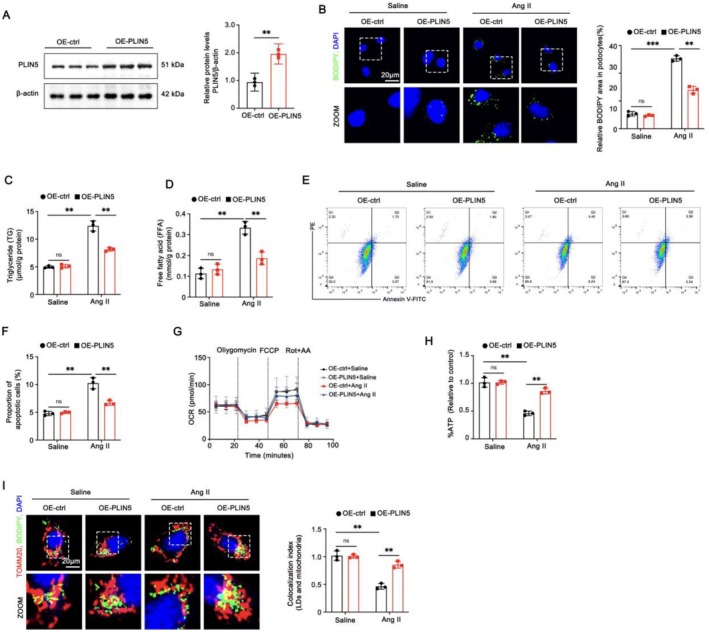
Overexpression of PLIN5 alleviated Ang II‐induced lipid dysregulation and lipid droplet–mitochondria contact disorder. (A) Verification of PLIN5 overexpression in cultured podocytes infected with control (OE‐ctrl) or PLIN5‐overexpressing (OE‐PLIN5) adenovirus, shown by Western blot and densitometric analysis (β‐actin as loading control). (B) Representative confocal images of neutral lipid droplets stained with BODIPY (green) and nuclei stained with DAPI (blue) in OE‐ctrl or OE‐PLIN5 podocytes treated with saline or Ang II and quantification of the relative BODIPY‐positive area. Scale bar, 20 μm. (C, D) Cellular triglyceride (TG) (C) and free fatty acid (FFA) (D) contents under the indicated conditions. (E, F) Representative Annexin V–FITC/PI flow‐cytometric plots (E) and quantification of the proportion of apoptotic podocytes (F). (G) Oxygen consumption rate (OCR) of podocytes measured by Seahorse analysis under basal conditions and after sequential injections of oligomycin, FCCP and rotenone/antimycin A (Rot + AA). (H) Intracellular ATP levels expressed as a percentage of saline‐treated OE‐ctrl cells. (I) Representative confocal images of podocytes stained for lipid droplets (BODIPY, green), mitochondria (TOMM20, red) and DAPI (blue) under the indicated treatments, with ZOOM views highlighting organelle contacts and quantification of the colocalisation index between lipid droplets and mitochondria. Scale bar, 20 μm. Data are presented as mean ± SEM from at least three independent experiments. ns, not significant; **p < 0.01, ***p < 0.001 (two‐way ANOVA with post hoc multiple comparisons).

To test whether PLIN5 protects podocytes from Ang II‐induced lipotoxicity and loss of lipid droplet‐mitochondria contact, cultured podocytes were transfected with PLIN5 pcDNA or control plasmid. Cells were then exposed to Ang II. Western blot analysis revealed that PLIN5 protein levels were significantly upregulated following transfection with the PLIN5 plasmid (Figure 5A). Moreover, PLIN5 overexpression reduced lipid deposition (Figure 5B–D), alleviated lipotoxicity (Figure 5E–H) and partially restored lipid droplet‐mitochondria contact (Figure 5I). Together with the preceding findings, these results confirm that PLIN5 protects podocytes against Ang II‐induced lipotoxicity and disruption of lipid droplet‐mitochondria contact.

### 3.6 Identification of FKBP8 as an Anchoring Site for PLIN5‐Mediated Lipid Droplet‐Mitochondria Contact

**FIGURE 6:**
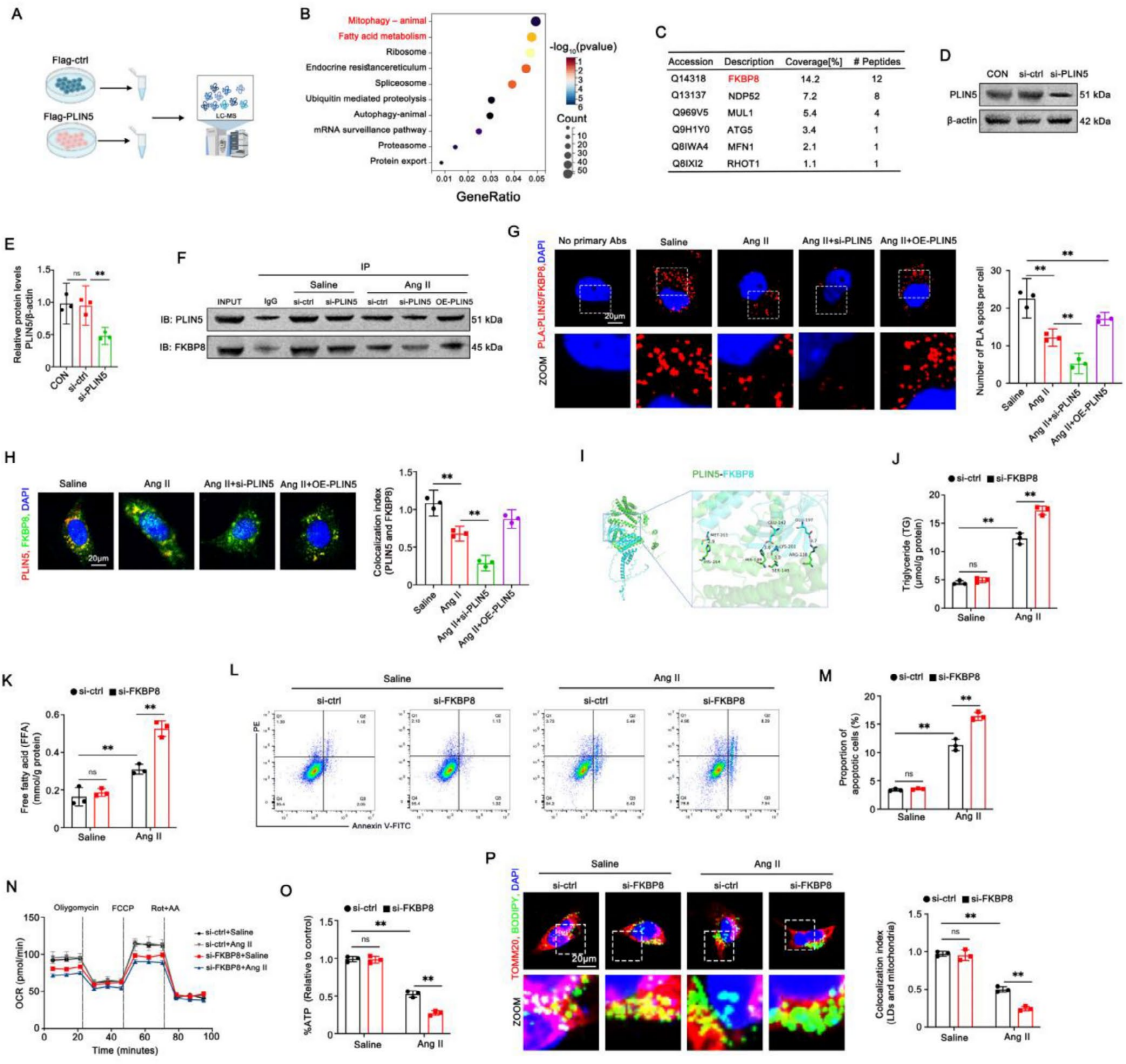
Identification of FKBP8 as an anchoring site for PLIN5‐mediated lipid droplet–mitochondria contact. (A) Schematic workflow of Flag‐PLIN5 immunoprecipitation coupled with LC–MS/MS to identify PLIN5‐associated proteins. (B) KEGG pathway enrichment analysis of PLIN5‐associated proteins identified by LC–MS/MS. Bubble plot shows the top enriched pathways; dot size represents the number of genes and colour indicates −log10 (*p* value). Mitophagy (animal) and fatty acid metabolism are highlighted in red. (C) List of representative PLIN5‐interacting proteins involved in mitophagy and mitochondrial quality control, including FKBP8 and NDP52, with protein accession number, sequence coverage and number of peptides identified. (D, E) Validation of PLIN5 knockdown efficiency in cultured podocytes transfected with control siRNA (si‐ctrl) or si‐PLIN5 by Western blot (D) and densitometric analysis (E; β‐actin as loading control). (F) Co‐immunoprecipitation (IP) analysis of the interaction between PLIN5 and FKBP8 in podocytes under the indicated conditions, followed by immunoblotting (IB) for PLIN5 and FKBP8. (G) Representative proximity ligation assay (PLA) images showing PLIN5‐FKBP8 interaction signals (red) in podocytes under the indicated conditions, including a no‐primary‐antibody negative control and enlarged ZOOM views. Nuclei were counterstained with DAPI (blue) and PLA puncta per cell were quantified from 30 to 50 cells per group in each of three independent experiments. Scale bar, 20 μm. (H) Representative immunofluorescence images showing PLIN5 (red), FKBP8 (green) and nuclei (DAPI, blue) in podocytes under the indicated conditions and quantification of the colocalisation index between PLIN5 and FKBP8. Scale bar, 20 μm. (I) Predicted structural model of the PLIN5‐FKBP8 complex, with a magnified view of the interaction interface showing key amino acid residues involved in binding. (J, K) Cellular triglyceride (TG; J) and free fatty acid (FFA; K) contents in podocytes transfected with si‐ctrl or si‐FKBP8 under saline or Ang II treatment. (L, M) Representative Annexin V‐FITC/PI flow‐cytometric plots (L) and quantification of the proportion of apoptotic podocytes (M) under the indicated conditions. (N) Oxygen consumption rate (OCR) traces in podocytes transfected with si‐ctrl or si‐FKBP8 under saline or Ang II treatment, measured by Seahorse analysis. (O) Intracellular ATP levels expressed as a percentage relative to control. (P) Representative confocal images of lipid droplets (BODIPY, green), mitochondria (TOMM20, red) and nuclei (DAPI, blue) in podocytes under the indicated conditions, with enlarged ZOOM views highlighting lipid droplet–mitochondria contact sites. Scale bar, 20 μm. Data are presented as mean ± SEM from at least three independent experiments. ns, not significant; **p < 0.01, ***p < 0.001 (one‐way or two‐way ANOVA with post hoc multiple comparisons, as appropriate).

Next, we sought to identify molecular partners that support lipid droplet–mitochondria interaction. We therefore performed Flag‐PLIN5 immunoprecipitation followed by LC–MS/MS analysis (Figure 6A,B). A representative FKBP8 peptide map from the mass spectrometry analysis is provided in Figure S2A. We further examined fatty acid oxidation and mitochondrial quality‐control markers to connect this proteomic finding with downstream metabolic changes. Ang II reduced CPT1α and altered ACC phosphorylation, whereas PLIN5 knockdown further aggravated these abnormalities and PLIN5 overexpression partially restored them; PINK1 and Parkin showed parallel changes in mitochondrial quality‐control signalling (Figure S2B,C). Among the candidate proteins enriched in pathways related to mitophagy and fatty acid metabolism, FKBP8 emerged as a, particularly, relevant candidate because of its localisation on the outer mitochondrial membrane (Figure 6C) [19]. PLIN5 knockdown efficiency was confirmed before interaction analysis (Figure 6D,E). Co‐IP assays showed that PLIN5 interacted with FKBP8 in podocytes (Figure 6F). To determine whether this interaction is altered under disease‐relevant conditions, we next examined the association between PLIN5 and FKBP8 in podocytes exposed to Ang II. Co‐IP showed that Ang II markedly reduced the amount of FKBP8 co‐precipitated with PLIN5, despite unchanged total FKBP8 abundance (Figure 6F). Consistently, PLA showed PLIN5‐FKBP8 puncta mainly in cytoplasmic and perinuclear regions, whereas the no‐primary‐antibody control produced minimal signals. Quantification of 30–50 cells per group from three independent experiments showed that Ang II reduced the number of PLA puncta, PLIN5 knockdown further decreased these signals and PLIN5 overexpression partially restored them (Figure 6G). Immunofluorescence staining with corrected channel labels (PLIN5, red; FKBP8, green; DAPI, blue) further confirmed reduced PLIN5‐FKBP8 colocalisation after Ang II treatment (Figure 6H,I). Together, these findings indicate that the interaction between these two proteins is dynamically impaired under lipotoxic stress.

In addition, Western blotting analysis showed that FKBP8 protein expression remained unchanged in kidney tissues from Ang II‐treated mice and in kidneys from PLIN5‐deficient mice (Figure S2D–E). Together, these findings indicate that FKBP8 is not regulated by Ang II or PLIN5 at the expression level, but rather participates in Ang II‐induced podocyte injury through altered interaction with PLIN5.

### 3.7 FKBP8 Is Functionally Required for Maintaining Lipid Droplet‐Mitochondria Contact and Limiting Ang II‐Induced Podocyte Lipotoxicity

Since the above findings suggested a disease‐relevant reduction in PLIN5‐FKBP8 interaction, we next asked whether FKBP8 is functionally required for lipid droplet‐mitochondria contact in podocytes. To this end, cultured podocytes were transfected with FKBP8‐specific siRNA prior to Ang II stimulation and knockdown efficiency was confirmed (Figure S2F). Under basal conditions, FKBP8 silencing caused only modest changes in intracellular lipid homeostasis. However, under Ang II stimulation, FKBP8 knockdown markedly aggravated neutral lipid accumulation, as demonstrated by increased TG and FFA contents (Figure 6J–O). Functionally, FKBP8‐deficient podocytes displayed more severe mitochondrial impairment after Ang II exposure, as reflected by lower OCR and reduced intracellular ATP levels (Figure 6N,O). In parallel, the proportion of apoptotic cells was significantly increased (Figure 6L,M). Furthermore, immunofluorescence colocalisation analysis showed that FKBP8 deficiency significantly reduced the overlap between lipid droplets and mitochondria in Ang II‐treated podocytes (Figure 6P), supporting a structural role for FKBP8 in maintaining organelle contact. Collectively, these data demonstrate that FKBP8 is functionally required for preserving lipid droplet–mitochondria contact and protecting podocytes against Ang II‐induced lipotoxic injury.

### 3.8 The 70–200 Amino Acid Region of FKBP8 Is Required for PLIN5 Binding and Organelle Contact Maintenance

**FIGURE 7:**
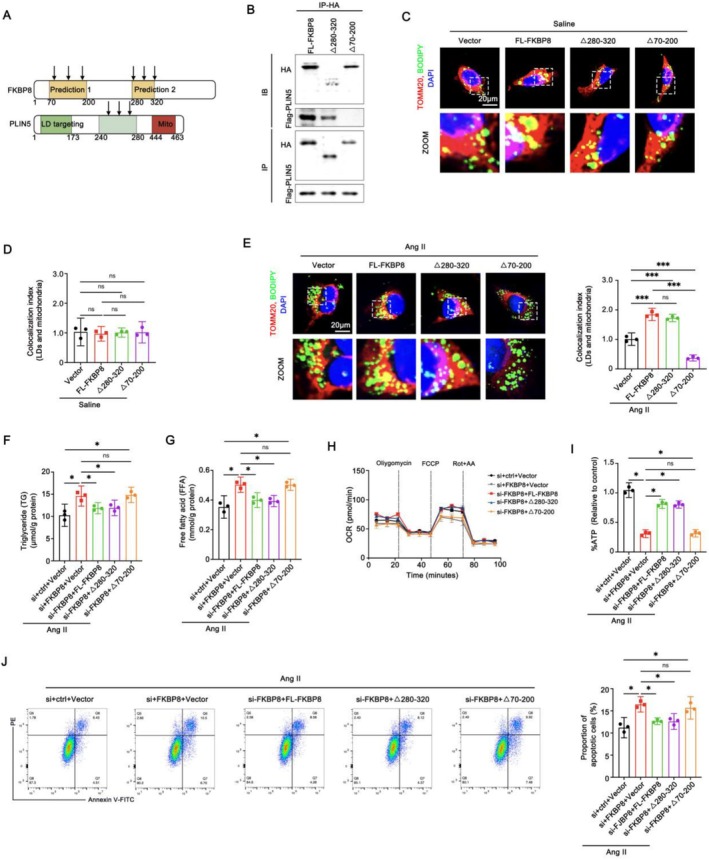
PLIN5 anchors to FKBP8 through its 70–200 amino acid domain. (A) Schematic representation of FKBP8 and PLIN5 protein domains. Two predicted PLIN5‐binding regions within FKBP8 (Prediction 1, 70–200 aa; Prediction 2, 280–320 aa) and the lipid droplet (LD)‐targeting and mitochondrial (Mito) regions of PLIN5 are indicated. (B) Co‐immunoprecipitation of Flag‐PLIN5 with HA‐tagged full‐length FKBP8 (FL‐FKBP8) or FKBP8 deletion mutants (Δ70–200, Δ280–320) in podocytes. Cell lysates were immunoprecipitated with anti‐HA antibody and analysed by Western blot (IB) for HA and Flag‐PLIN5. (C) Representative confocal images of podocytes transfected with empty vector, FL‐FKBP8, FKBP8‐Δ280Δ320 or FKBP8‐Δ70–200 under saline treatment. Lipid droplets were labelled with BODIPY (green), mitochondria with TOMM20 (red) and nuclei with DAPI (blue). ZOOM panels show enlarged regions outlined by dashed boxes. Scale bar, 20 μm. (D) Quantification of the colocalisation index between lipid droplets and mitochondria in podocytes under saline treatment. (E) Representative confocal images of podocytes transfected with empty vector, FL‐FKBP8, FKBP8‐Δ280–320 or FKBP8‐Δ70–200 under Ang II treatment, with ZOOM views and quantification of the colocalisation index between lipid droplets and mitochondria. Scale bar, 20 μm. (F, G) Cellular triglyceride (TG; F) and free fatty acid (FFA; G) contents in Ang II‐treated podocytes transfected with si‐ctrl+Vector, si‐FKBP8 + Vector, si‐FKBP8 + FL‐FKBP8, si‐FKBP8 + Δ280–320 or si‐FKBP8 + Δ70–200. (H) Oxygen consumption rate (OCR) traces in Ang II‐treated podocytes under the indicated rescue conditions, measured by Seahorse analysis. (I) Intracellular ATP levels expressed as a percentage relative to control in Ang II‐treated podocytes under the indicated rescue conditions. (J) Representative Annexin V‐FITC/PI flow‐cytometric plots and quantification of the proportion of apoptotic podocytes under the indicated rescue conditions in the presence of Ang II. Data are presented as mean ± SEM from at least three independent experiments. ns, not significant; **p < 0.01, ***p < 0.001 (one‐way ANOVA with post hoc multiple comparisons).

To further elucidate the anchoring mechanism between PLIN5 and FKBP8, we used computational modelling to predict their potential binding regions. The results suggested that PLIN5 may bind FKBP8 through either the 70–200 amino acid region or the 280–320 amino acid region (Figure 7A). To verify this prediction, we generated HA‐tagged full‐length FKBP8 (FKBP8‐FL), FKBP8‐Δ70–200 and FKBP8‐Δ280–320 constructs. Co‐IP experiments demonstrated that PLIN5 bound robustly to full‐length FKBP8 and the Δ280–320 mutant, whereas deletion of the 70–200 amino acid region markedly impaired PLIN5 binding (Figure 7B).

To further define the pathological relevance of this domain, we expanded the analysis by including both basal and Ang II‐stimulated conditions. Under saline treatment, expression of FKBP8‐FL or FKBP8‐Δ280–320 had little effect on lipid droplet‐mitochondria colocalisation, whereas FKBP8‐Δ70–200 showed a mild but detectable reduction in organelle contact (Figure 7C,D). Under Ang II stimulation, however, the difference became much more evident: full‐length FKBP8 and the Δ280–320 construct significantly restored lipid droplet‐mitochondria colocalisation, whereas the Δ70–200 mutant failed to do so (Figure 7E).

In agreement with these findings, Ang II‐treated podocytes expressing FKBP8‐Δ70–200 exhibited significantly higher TG and FFA levels than those expressing full‐length FKBP8 or FKBP8‐Δ280–320 (Figure 7F,G). Moreover, the Δ70–200 mutant failed to suppress Ang II‐induced ATP depletion and apoptosis (Figure 7H–J). These data indicate that the 70–200 amino acid region of FKBP8 is structurally and functionally required for PLIN5 binding and for maintenance of lipid droplet–mitochondria contact under lipotoxic stress.

### 3.9 The Protective Effects of PLIN5 Overexpression Against Ang II‐Induced Lipid Accumulation and Lipotoxicity in Podocytes In Vivo

**FIGURE 8:**
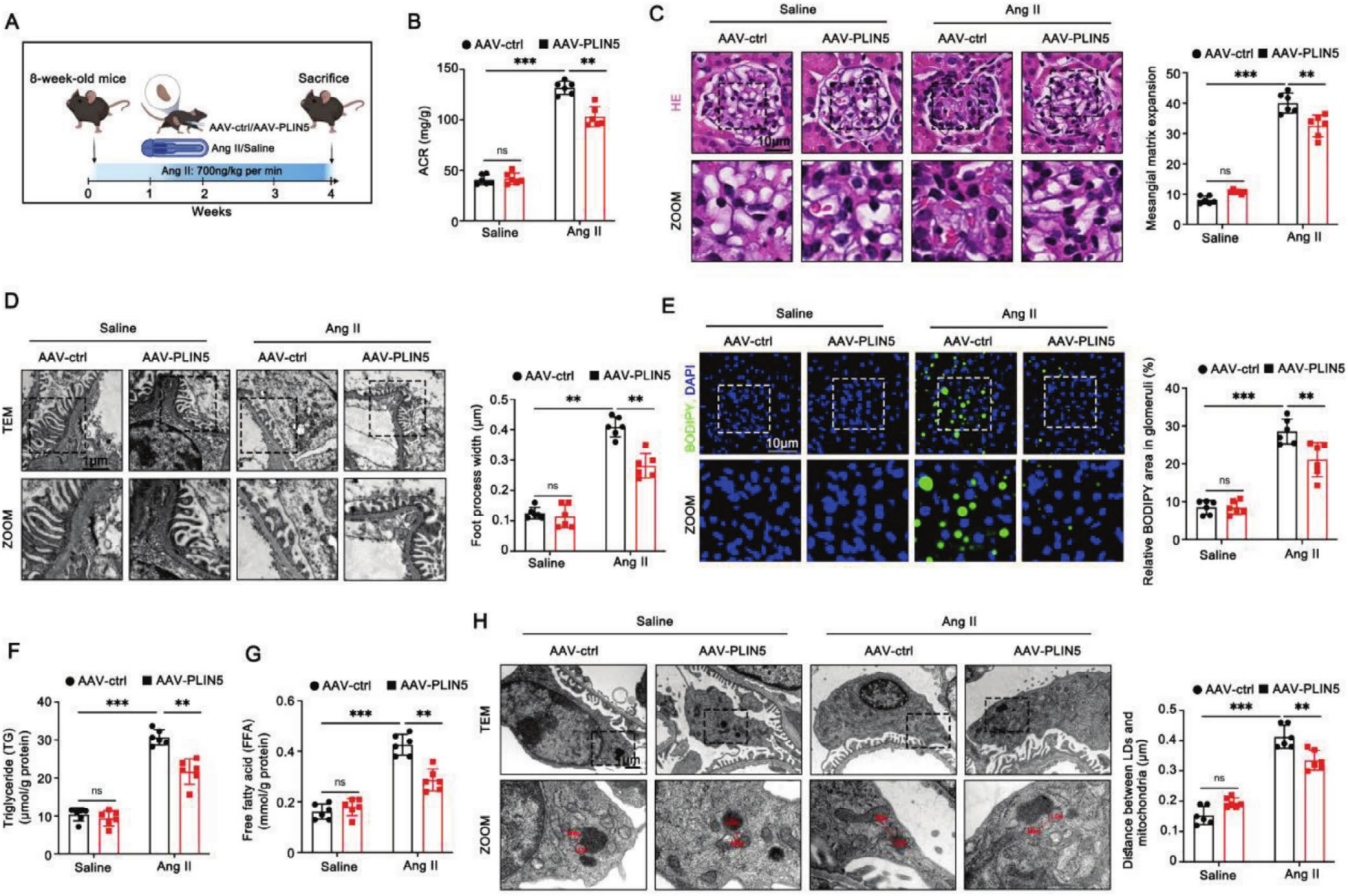
The protective effects of PLIN5 overexpression against Ang II‐induced lipid accumulation and lipotoxicity in podocytes in vivo. (A) Experimental scheme. Eight‐week‐old mice were injected with AAV carrying control vector (AAV‐ctrl) or PLIN5 (AAV‐PLIN5), followed by continuous infusion of Ang II (700 ng/kg per min) or saline for 4 weeks before sacrifice. (B) Urinary albumin‐to‐creatinine ratio (ACR) in the indicated groups. (C) Representative HE‐stained kidney sections showing glomerular morphology in AAV‐ctrl and AAV‐PLIN5 mice with or without Ang II infusion and quantification of mesangial matrix expansion. Scale bar, 10 μm. (D) Representative transmission electron microscopy (TEM) images of podocyte foot processes and quantification of foot process width. Scale bar, 1 μm. (E) Representative images of neutral lipid accumulation in glomeruli detected by BODIPY (green) with DAPI (blue) and quantification of the relative BODIPY‐positive area. (F, G) Renal triglyceride (TG) (F) and free fatty acid (FFA) (G) contents in the indicated groups. (H) Representative TEM images showing lipid droplets (LDs) and mitochondria (Mt) in podocytes and quantification of the mean distance between LDs and mitochondria. Scale bar, 1 μm. Data are presented as mean ± SEM (*n* = 6 mice per group). ns, not significant; **p < 0.01, ***p < 0.001 (two‐way ANOVA with post hoc multiple comparisons).

Given that PLIN5 has been implicated in protection against Ang II‐induced lipotoxicity and impairment of lipid droplet‐mitochondria contact in podocytes, AAV9‐PLIN5 was administered via glass micropipette injection to mice to further validate the mechanism of PLIN5 in vivo. The mice in the experimental group were infused with Ang II, while those in the control group received physiological saline. The results showed that PLIN5 protein levels were significantly upregulated after AAV9 injection (Figure 8A) and Ang II‐induced increases in urine ACR, mesangial cell proliferation and effacement of podocyte foot processes in the glomeruli were reversed (Figure 8B–D). Furthermore, Ang II‐induced lipid accumulation in the glomeruli and synaptopodin‐positive podocytes were alleviated (Figure 8E–G). Additionally, the impairment of lipid droplet‐mitochondria contact induced by Ang II was also mitigated (Figure 8H). Thus, these findings demonstrate the protective role of PLIN5 against Ang II‐induced podocyte lipid accumulation and subsequent kidney injury, indicating that PLIN5 holds promise as a potential therapeutic agent for podocytopathy.

## 4 Discussion

Dysregulated lipid metabolism leads to mitochondrial dysfunction and cellular damage in kidney cells, particularly podocytes, thereby promoting the progression of kidney injury [20]. In recent years, research into the pathogenesis of podocyte lipotoxicity has garnered increasing attention [21, 22]. Building on our previous work demonstrating the critical role of Ang II‐induced podocyte lipotoxicity [15], this study identifies PLIN5 as a central regulator in this pathogenic process. We show that PLIN5 expression is significantly reduced in podocytes under Ang II stimulation both in vitro and in vivo and that genetic or experimental reduction of PLIN5 aggravates lipid accumulation, mitochondrial dysfunction and apoptosis. Conversely, PLIN5 overexpression ameliorates these abnormalities. More importantly, this study further demonstrates that FKBP8 is functionally required for this protective effect. Ang II weakens the interaction between PLIN5 and FKBP8; FKBP8 deficiency disrupts lipid droplet‐mitochondria contact and aggravates podocyte lipotoxicity and re‐expression of wild‐type FKBP8, but not the FKBP8‐Δ70–200 mutant, rescues these defects. Together, these findings support a model in which PLIN5 protects podocytes, at least in part, by interacting with FKBP8 and preserving lipid droplet‐mitochondria contact under lipotoxic stress.

PLIN5, a lipid droplet surface protein, serves as a key regulator in lipid homeostasis [9]. First, PLIN5 maintains the structural integrity of lipid droplets by interacting with adipose triglyceride lipase (ATGL) and its coactivator, comparative gene identification‐58 (CGI‐58), thereby preventing premature activation of lipolysis [23, 24]. Second, as a structural component of the membrane contact site complex, PLIN5 facilitates the exchange of molecules and signals between lipid droplets and mitochondria [12]. Third, PLIN5 enhances mitochondrial biogenesis, elevates the activity of mitochondrial enzymes and promotes fatty acid oxidation [25]. Downregulation or specific knockout of PLIN5 results in reduced lipid droplet‐mitochondria contact, decreased mitochondrial content and the accumulation of free lipids within cells. These alterations subsequently trigger inflammatory responses, endoplasmic reticulum stress and lipid peroxidation‐induced damage [13]. Recently, Miner et al. demonstrated that PLIN5 could interact with the acyl‐CoA synthetase fatty acid transport protein 4 (FATP4) on mitochondria to facilitate fatty acid transport and oxidation in starved myoblasts. This interaction promoted the intracellular trafficking of free lipids from lipid droplets to mitochondria, enhancing mitochondrial lipid utilisation and protecting cells from lipotoxic damage caused by the accumulation of free lipids [26]. While its role in facilitating lipid droplet‐mitochondria crosstalk has been recognised, the specific molecular tethers in podocytes remain unknown. Our study addresses this gap by identifying FKBP8 on the outer mitochondrial membrane as a novel binding partner for PLIN5. These findings suggest that PLIN5 may participate in multiple molecular interactions and exert diverse biological functions. Therefore, further investigation into the functional roles of PLIN5 is warranted.

FKBP8 is a member of the FK506‐binding protein family and is localised primarily on the outer mitochondrial membrane [27]. Previous studies have implicated FKBP8 in mitochondrial quality control and in communication with other organelles, including the endoplasmic reticulum and lysosomes [28, 29]. FKBP8 was identified here as a PLIN5‐interacting protein in podocytes and was found to be required for maintenance of lipid droplet–mitochondria contact under Ang II stress. Notably, neither Ang II treatment nor PLIN5 deficiency altered total FKBP8 abundance, suggesting that FKBP8 contributes to Ang II‐induced podocyte lipotoxicity primarily through altered protein interaction and organelle‐contact function rather than through changes in expression. These findings indicate that disruption of the PLIN5‐FKBP8 complex, rather than downregulation of FKBP8 itself, is a disease‐relevant event in podocyte lipotoxic injury.

Notably, our data show that FKBP8 was functionally required, rather than merely associated, in the PLIN5‐dependent protective pathway. Loss of FKBP8 recapitulated the detrimental effects of PLIN5 deficiency, including reduced organelle contact, impaired mitochondrial respiration, ATP depletion, ROS accumulation and enhanced apoptosis. By contrast, re‐expression of wild‐type FKBP8 restored these defects. Together, these observations support the notion that FKBP8 serves as a critical functional node through which PLIN5 helps preserve lipid metabolic homeostasis in podocytes [26].

Another important finding is that the 70–200 amino acid region of FKBP8 functions as a key domain for PLIN5 binding and protection against lipotoxic stress. Deletion of this region markedly weakened PLIN5 binding, reduced lipid droplet–mitochondria contact and abolished the protective effect of FKBP8 in Ang II‐treated podocytes. In contrast, deletion of the 280–320 region had comparatively little impact. These data identify the 70–200 aa region as a structurally important domain for the PLIN5–FKBP8 interaction. Although this region may represent a candidate interface for future mechanistic investigation or drug discovery, the current data support its role primarily as a functionally relevant binding domain rather than a validated therapeutic target.

Proteomic enrichment analysis highlighted pathways related to mitochondrial homeostasis and mitophagy, raising the possibility that PLIN5‐interacting proteins may affect multiple aspects of mitochondrial regulation [27, 28]. Consistent with this possibility, disruption of the PLIN5‐FKBP8 axis was associated with impaired fatty acid utilisation, as reflected by reduced CPT1α expression and altered ACC phosphorylation, together with moderate changes in mitophagy‐related markers such as PINK1, Parkin, LC3 and p62 [30]. These findings suggest that disturbed organelle contact may have broader consequences for mitochondrial quality control [27, 28, 31, 32]. However, impaired lipid handling and mitochondrial bioenergetics appeared to be the dominant downstream events in this model [30]. Taken together, these results support a model in which defective lipid droplet–mitochondria communication compromises fatty acid utilisation and sensitises podocytes to lipotoxic injury, rather than indicating that the PLIN5‐FKBP8 axis primarily regulates mitophagy [26, 30].

Given that FKBP8 belongs to the FK506‐binding protein family, it is conceivable that pharmacological agents targeting this family may influence organelle‐contact regulation in podocytes [33, 34]. However, the effect of tacrolimus or other FK506‐binding agents on the PLIN5‐FKBP8 complex was not examined here. Therefore, any connection between FKBP8‐targeting drugs and podocyte lipid homeostasis remains speculative and will require direct experimental evaluation in future studies.

The present study has several limitations. First, although we provide functional evidence that FKBP8 is required for maintaining lipid droplet‐mitochondria contact in cultured podocytes, additional in vivo studies specifically targeting FKBP8 in podocytes would further strengthen the physiological relevance of this mechanism. Second, while our data show that Ang II weakens the interaction between PLIN5 and FKBP8, the upstream molecular events responsible for this disruption remain unclear. For example, stress‐induced post‐translational modifications of PLIN5 or FKBP8 may alter their binding affinity and deserve further investigation. Third, although changes in fatty acid metabolism and mitochondrial quality‐control markers accompanied disruption of the PLIN5‐FKBP8 axis, the precise causal relationship between organelle contact, fatty acid utilisation and mitophagy requires more detailed mechanistic dissection. Finally, the structural basis by which the 70–200 aa region of FKBP8 mediates PLIN5 binding remains to be resolved at higher resolution.

In conclusion, this study reveals a protective role of PLIN5 against Ang II‐induced podocyte lipotoxicity and identifies FKBP8 as a functionally important interacting partner in this process. These findings support a model in which disruption of the PLIN5‐FKBP8 complex under Ang II stress impairs lipid droplet–mitochondria contact, compromises fatty acid utilisation and mitochondrial bioenergetics and thereby promotes podocyte injury. Furthermore, the 70–200 amino acid region of FKBP8 is required for PLIN5 binding and for the protective effect of this axis. These findings provide mechanistic insight into how organelle communication regulates podocyte lipid homeostasis and highlight the PLIN5‐FKBP8 interaction as a potential target for future investigation in lipotoxic kidney disease.

## Author Contributions

**Ping Wang:** writing – review and editing, project administration, funding acquisition. **Wenjie Chen:** review and editing, project administration, conceptualisation. **Jingjing Ke:** validation, methodology. **Hongtu Hu:** validation, methodology. **Zhuan Peng:** validation, methodology. **Yue Qi:** validation, methodology. **Guohua Ding:** writing – review and editing, supervision, project administration, funding acquisition, conceptualisation. **Jijia Hu:** writing – review and editing, validation, methodology, investigation, supervision, project administration, funding acquisition.

## Funding

This work was supported by the National Natural Science Foundation of China (82300767, 82260150 and 82070713).

## Ethics Statement

In this study, animal experiments were granted by the Ethical Committee for the Experimental Use of Animals at Renmin Hospital, Wuhan University, China (WDRM‐20220205). All mice received humane care and all mouse experiments complied with National Institutes of Health (NIH) regulations.

## Consent

The authors have nothing to report.

## Conflicts of Interest

The authors declare no conflicts of interest.

## Supporting information


**Figure S1:** Construction and validation of PLIN5^podKO^ mice. Related to Figure 3.
**Figure S2:** FKBP8 peptide map and validation of downstream metabolic and FKBP8‐expression analyses.
**Table S1:** Primary antibodies used in the experiments.

**Data S1:** cpr70257‐sup‐0002‐Supinfo02.jpg.

## Data Availability

The data that support the findings of this study are available from the corresponding author upon reasonable request.
