# Supplementary material for: PLIN5 Protects Against Ang II‐Induced Podocyte Lipotoxicity by Interacting With FKBP8 and Preserving Lipid Droplet–Mitochondria Contact
Source: Cell Prolif. 2026 Jun 30:e70257. Online ahead of print. doi: 10.1111/cpr.70257 (PMC13325938; doi:10.1111/cpr.70257)
Supplement: Supplementary file 1 — Figure S1: Construction and validation of PLIN5podKO mice. Related to Figure 3. Figure S2: FKBP8 peptide map and validation of downstream metabolic and FKBP8‐expression analyses. Table S1: Primary antibodies used in the experiments. [file CPR-9999-e70257-s002.docx]

**Supplementary Figure**

**
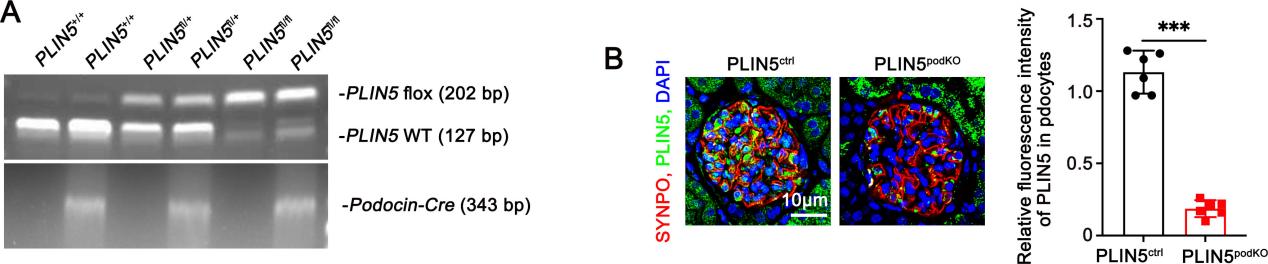
**

**Figure S1. Construction and Validation of PLIN5^podKO^ Mice. Related to Figure 3.**

(**A**) Genotyping the mice by PCR analysis of genomic DNA; lanes 1-5 are PLIN5^ctrl^ genotype and lane 6 is PLIN5^podKO^ genotype. (**B**) Representative confocal images of glomeruli co-stained for synaptopodin (SYNPO, red) and PLIN5 (green) with DAPI (blue) and quantification of the relative fluorescence intensity of PLIN5 in SYNPO-positive podocytes. Scale bar, 10 μm. n=6 mice per group. *** P < 0.001.


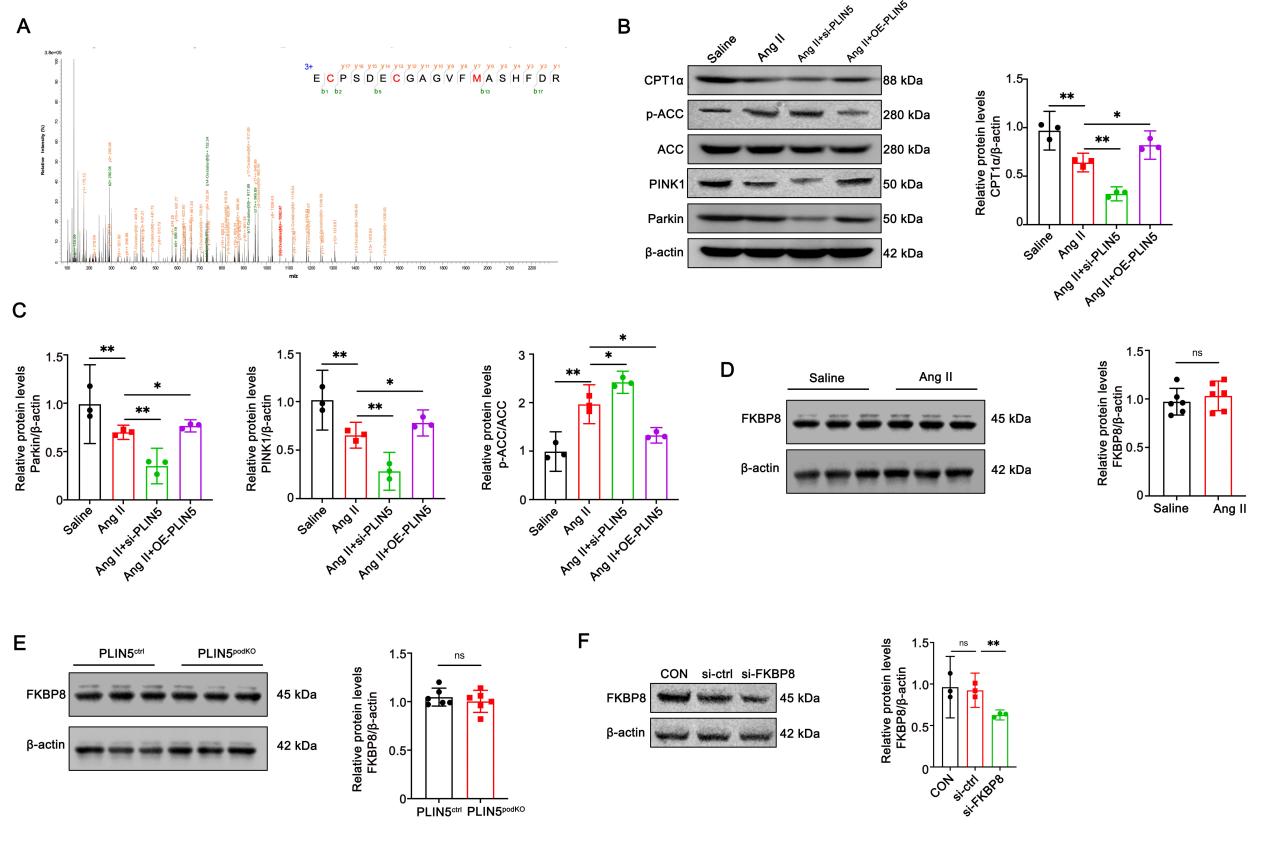


**Figure S2. FKBP8 peptide map and validation of downstream metabolic and FKBP8-expression analyses.** (A) Representative LC-MS/MS peptide map for FKBP8 identified in the PLIN5 immunoprecipitation sample. (B, C) Representative Western blots and densitometric analyses of CPT1α, Parkin, PINK1, and p-ACC/ACC in cultured podocytes treated with saline, Ang II, Ang II plus si-PLIN5, or Ang II plus OE-PLIN5. (D) Representative Western blot and quantification of FKBP8 protein levels in kidney tissues from saline- and Ang II-treated mice. (E) Representative Western blot and quantification of FKBP8 protein levels in kidney tissues from PLIN5^ctrl^ and PLIN5^podKO^ mice. (F) Representative Western blot and densitometric analysis confirming FKBP8 knockdown efficiency in cultured podocytes transfected with si-FKBP8. ns, not significant; *P < 0.05, **P < 0.01 (one-way or two-way ANOVA with post hoc multiple comparisons, as appropriate).

**Supplementary Table**

**Table 1. Primary antibodies used in the experiments.**

| **Antigen** | **Application** | **Dilution** | **Source** | **Catalog no.** |
| --- | --- | --- | --- | --- |
| PLIN5 | WB | 1:2000 | Proteintech | 26951-1-AP |
| FKBP8 | WB | 1:1000 | Thermo Fisher Scientific | MA5-35702 |
| β-actin | WB | 1:5000 | Proteintech | 20536-1-AP |
| CPT1α | WB | 1:1000 | Cell Signaling Technology | 12252 |
| Phospho-ACC (Ser79) | WB | 1:1000 | Cell Signaling Technology | 3661 |
| ACC | WB | 1:1000 | Cell Signaling Technology | 3662 |
| PINK1 | WB | 1:1000 | Cell Signaling Technology | 6946 |
| Parkin | WB | 1:1000 | Cell Signaling Technology | 2132 |
| TOMM20 | IF | 1:200 | Cell Signaling Technology | 42406 |
| WT1 | IF | 1:100 | Boster | M00199-1 *(alternative code: BM4216)* |
| Synaptopodin | IF | 1:200 | Proteintech | 20384-1-AP |
| HA tag | WB / Co-IP | 1:1000 | Sigma-Aldrich | H3663 |
| FLAG tag | WB / Co-IP | 1:1000 | Sigma-Aldrich | F1804 |
