# Supplementary figures and images for: PLIN5 Protects Against Ang II‐Induced Podocyte Lipotoxicity by Interacting With FKBP8 and Preserving Lipid Droplet–Mitochondria Contact
Source: Cell Prolif. 2026 Jun 30:e70257. Online ahead of print. doi: 10.1111/cpr.70257 (PMC13325938; doi:10.1111/cpr.70257)

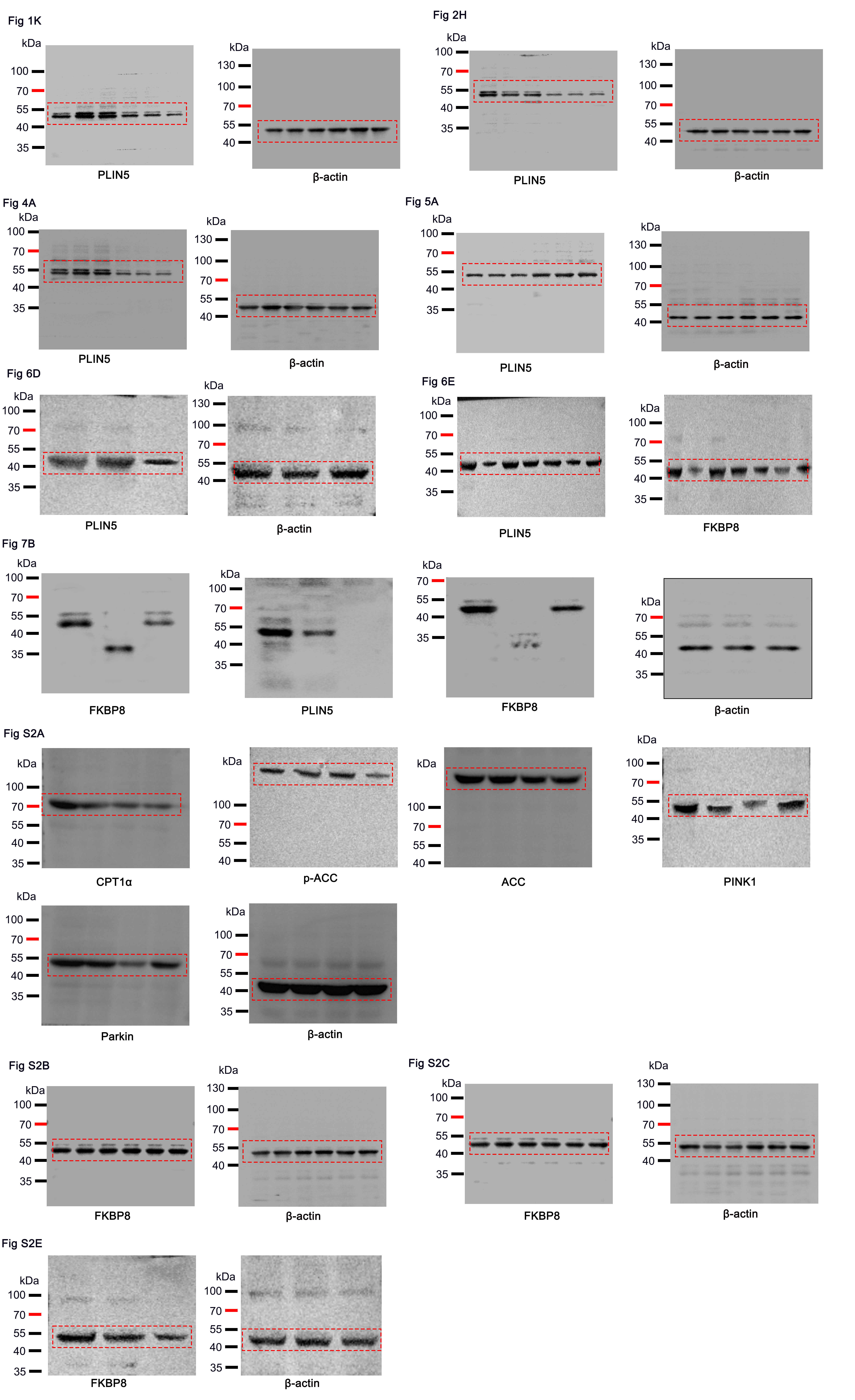

Supplement: Supplementary file 2 — Data S1: cpr70257‐sup‐0002‐Supinfo02.jpg. [file CPR-9999-e70257-s001.jpg]
